# Implementação Clínica de Diferentes Estratégias para Reabilitação Baseada em Exercícios em Receptores de Transplante de Rim e Fígado: Um Estudo Piloto

**DOI:** 10.36660/abc.20210159

**Published:** 2022-07-29

**Authors:** Paula A. B. Ribeiro, Mathieu Gradassi, Sarah-Maude Martin, Jonathan Leenknegt, Mathilde Baudet, VyVan Le, Marie-Pascale Pomey, Agnes Räkel, François Tournoux

**Affiliations:** 1 Centre Hospitalier de l’Université de Montréal Centre de Recherche Unité de recherche Quebec Canadá Unité de recherche @coeurlab – Centre de Recherche du Centre Hospitalier de l’Université de Montréal (CRCHUM), Quebec – Canadá; 2 Centre Hospitalier de l’Université de Montréal Centre de Cardiologie Preventive Quebec Canadá Centre de Cardiologie Preventive du Centre Hospitalier de l’Université de Montréal, Quebec – Canadá; 3 Université du Québec à Montréal Département des sciences de l’activité physique Québec Canadá Département des sciences de l’activité physique, Université du Québec à Montréal, Québec – Canadá; 4 Centre Hospitalier de l’Université de Montréal Département de Cardiologie Québec Canadá Département de Cardiologie du Centre Hospitalier de l’Université de Montréal, Québec – Canadá; 5 Université de Montréal École de santé publique Québec Canadá École de santé publique, Université de Montréal, Québec – Canadá; 6 Centre Hospitalier de l’Université de Montréal Département d’Encrinologie Québec Canadá Département d’Encrinologie du Centre Hospitalier de l’Université de Montréal, Québec – Canadá

**Keywords:** Exercício, Técnicas de Exercício e Movimento, Condicionamento Físico humano, Rim/transplante, Fígado/transplante, Terapia por Exercício

## Abstract

**Fundamento:**

A doença cardiovascular está entre as principais causas de morte entre pacientes transplantados. Embora esses pacientes possam teoricamente se beneficiar de programas de reabilitação baseada em exercícios (RBE), sua implementação ainda é um desafio.

**Objetivo:**

Apresentamos nossa experiência inicial em diferentes modos de realização de um programa piloto de RBE em receptores de transplante de rim e fígado.

**Métodos:**

Trinta e dois pacientes transplantados renais ou hepáticos foram convidados para um programa de RBE de 6 meses realizado na academia do hospital, na academia comunitária ou em casa, de acordo com a preferência do paciente. O nível de significância adotado foi de 5%.

**Resultados:**

Dez pacientes (31%) não completaram o programa. Entre os 22 que completaram, 7 treinaram na academia do hospital, 7 na academia comunitária e 8 em casa. O efeito geral foi um aumento de 11,4% nos METs máximos (tamanho do efeito de Hedges g = 0,39). O grupo de academia hospitalar teve um aumento nos METs de 25,5% (g = 0,58, tamanho de efeito médio) versus 10% (g = 0,25) e 6,5% (g = 0,20) para os grupos de academia comunitária e em casa, respectivamente. Houve efeito benéfico nas pressões arteriais sistólica e diastólica, maior para os grupos academia hospitalar (g= 0,51 e 0,40) e academia comunitária (g= 0,60 e 1,15) do que para os pacientes treinando em casa (g= 0,07 e 0,10). Nenhum evento adverso significativo foi relatado durante o seguimento.

**Conclusão:**

Programas de RBE em receptores de transplante de rim e fígado devem ser incentivados, mesmo que sejam realizados fora da academia do hospital, pois são seguros com efeitos positivos na capacidade de exercício e nos fatores de risco cardiovascular.

## Introdução

A sobrevida a curto prazo entre os receptores de transplante de órgãos sólidos (RTOS) melhorou significativamente devido à diminuição da mortalidade por infecções e rejeições agudas de orgãos.^1^ Embora os receptores de transplantes de fígado e rim tenham um risco cardiovascular (CV) menor do que seus pares em lista de espera de transplante,^2,3^ seu risco de mortalidade ainda é maior do que a população geral.^4,5^ De fato, as doenças cardiovasculares são as causas mais comuns de morte em pacientes transplantados e são responsáveis por 30% da perda precoce do orgão após o transplante renal.^4,6^ Certos fatores de risco pré-transplante, incluindo diabetes, hipertensão, dislipidemia e obesidade, contribuem para esse alto risco CV.^6,7^ Há também fatores pós-transplante que contribuem para esse risco CV, como novo aparecimento de diabetes,^8^ desenvolvimento de síndrome metabólica^9^ e sedentarismo.^10^ A maioria dos RTOSs não atinge os níveis de atividade física recomendados pelas diretrizes em sua rotina diária^11,12^ sugerindo que os pacientes poderiam se beneficiar de mais orientações e suporte social e profissional personalizado^13,14^ para melhorar sua atividade física diária.

Uma vez que os programas de reabilitação baseados em exercícios (RBE) melhoram os fatores de risco cardiovascular na população geral,^6,15^ espera-se que tenham um impacto benéfico em receptores de transplante de órgãos sólidos. Embora os efeitos desses programas sejam bem conhecidos em receptores de transplante cardíaco e pulmonar (devido aos efeitos diretos do exercício na função cardíaca e pulmonar),^16–19^ seus benefícios e segurança são mais incertos para outros pacientes com RTOS.^17,18,20,21^ Custos, logística e cobertura do seguro também são barreiras significativas^22^ que limitam a implementação rápida e ampla desses programas de RBE para essa população específica. Acreditamos que estratégias de realização personalizadas e programas externos ao ambiente clínico podem ser úteis para superar esses desafios, assim como, capitar pacientes que nao participariam de programas realizados em hospitais, especialmente em situações inesperadas, como a pandemia de COVID-19.^23,24^

Portanto, apresentamos nossa experiência inicial sobre os efeitos cardiovasculares de diferentes modos de realização de um programa piloto de RBE em receptores de transplante de rim e fígado.

## Métodos

Em 2016, realizamos em nossa instituição um estudo piloto randomizado sobre o impacto do treinamento de resistência sobre os fatores envolvidos no desenvolvimento de diabetes de início recente após transplante renal.^20^ Aprendemos com este estudo que quase 55% de nossos pacientes recusaram o convite para participar porque não puderam vir ao nosso centro com a frequência exigida pelo programa (3 vezes por semana). Nossa equipe decidiu projetar um novo programa de RBE para pacientes com transplante de rim e fígado, que pode ser entregue na academia do hospital, na academia comunitária ou em casa, dependendo da preferência do paciente. Apresentamos aqui nossa experiência inicial com os primeiros 32 pacientes envolvidos neste novo programa de RBE. Esta análise retrospectiva foi aprovada pelo Comitê de Ética em Pesquisa do CRCHUM, que está em conformidade com a Declaração de Helsinki (REC 2017-6733).

### O programa RBE

Em nossa instituição, os RTOS (18 anos ou mais) são convidados a ingressar no programa RBE após o transplante como parte de sua trajetória assistencial, geralmente 6 meses após o transplante renal e 9 meses após o transplante hepático. Todos os pacientes receptores de rim e fígado que participaram de nosso programa entre 2016 e 2018 foram incluídos em nossa análise. A avaliação pré-participação (exame físico e teste de esforço) foi realizada no hospital por um cardiologista e um cinesiologista. Na ausência de contraindicação cardiovascular, cada paciente participou de um RBE de 6 meses, adaptado de acordo com sua condição atual e consultado se preferia ou não treinar fora do contexto hospitalar. Na ocasião foi feita uma discussão entre o paciente e o cinesiologista sobre os prós e contras. A prescrição do exercício seguiu as recomendações do ACSM e CAN-Restore,^25,26^ combinando exercícios aeróbicos, resistidos e de flexibilidade: 1) treinamento aeróbico: 3-5 vezes por semana, visando 50-80% VO_2_max (5-6 Borg), iniciando com 20 min/seção e aumentando progressivamente até 60 min; 2) treinamento de resistência: 2-3 vezes por semana, 1-3 séries de 10 a 15 repetições de 5-6 exercícios (total de 20 a 30 min), utilizando exercícios multiarticulares incluindo os principais grupos musculares de acordo com as habilidades do paciente (a lista completa dos exercícios prescritos está disponível no material complementar – Tabela 1); e 3) exercícios de flexibilidade 2-3 vezes por semana, 2-3 exercícios/posições de acordo com a limitação pelos sintomas do paciente (ou seja, dor). A tabela de prescrição está disponível no material complementar – Tabela 2.

**Tabela 1:** Características clínicas de acordo com o grupo de intervenção

		Academia hospitalar (n=7)	Academia comunitária (n=7)	Em casa (n=8)	TOTAL (n=22)
Idade		58,0±6,9	53,7±12	60,4±8,0	57,5±9,2
Gênero (M/F)		5/2	3/4	6/2	14/8
Tempo de transplante (meses)		126±97	103±71	113±93	114±84
Intervalo de tempo de transplante (min-max)		253 (5 - 258)	198 (8 - 206)	242 (12 – 254)	253 (5 - 258)
**Transplante (n)**					
	Rim	2	6	7	15
	Fígado	3	1	0	4
	Rim+Pâncreas	2	0	1	3
Diabetes		2	5	3	10
Hipertensão		4	5	6	15
**Uso de medicamentos**					
	Bloqueador beta	2	6	3	11
	Imunossupressores	4	4	7	15

Os valores são apresentados como média ± DP ou número de pacientes (porcentagens); GLM: modelo linear generalizado;

* diferença de grupo (HOSPITAL vs CASA): p=0,017.

**Tabela 2:** Características clínicas de acordo com o grupo de intervenção

		Academia hospitalar (n=7)		Academia comunitária (n=7)		Em casa (n=8)		TOTAL (n=22)		Interação GLM
		Pré	Pós	Pré	Pós	Pré	Pós	Pré	Pós	
Peso (Kg)		81,3±18,9	81,3±20,4	91,4±14,7	85,3±15,1	82,9±12,0	80,3±13,9	85,1±15,2	82,1±16,2	0,87
IMC (m/kg^2^)		28,6±5,8	28,6±6,4	32,1±4,8	30,1±2,5	30,1±4,7	29,1±4,6	30,2±5,1	29,2±4,7	0,86
Circunferência da cintura (cm)		100,4±15,5	98,9±16,1	111,8±11,7	105,0±8,6	105,7±8,4	105,0±9,2	105,7±12,7	102,6±12,2	0,78
**Teste de esforço**										
	METs máx	5,5±2,3	6,9±2,2	6,0±2,0	6,6±2,4	6,1±1,7	6,5±2,0	5,8±1,9	6,6±2,1	0,76
	METs previstos (%)	75±28	96±31	81±34	87±37	91±35	96±40	82±32	93±35	0,76
	VO_2_máx calculado (ml.kg.min^−1^)	19,2±7,9	24,1±7,8	21,1±7,0	23±8,3	21,2±6,0	22,8±7,1	20,5±6,7	23,3±7,4	0,76
	Tempo de exercício (min)	7:47±3:51	8:11±3:21	6:00±1:31	7:00±1:37	7:37±2:36	7:30±2:55	7:09±2:47	7:33±2:39	0,86
	FC máx (bpm)	133±18	131±35	131±33	130±35	131±26	130±25	132±25	130±30	0,99
	FC previsto (%)	82±12	80±23	78±20	77±19	80±14	81±17	80±15	79±18	0,98
	PAS pré-teste	131±15	122±18	138±20	127±14	125±16	124±9	131±17	124±10	0,55
	PAD pré-teste	74±8	71±6	81±6	73±7	76±8	75±10	77±8	73±7	0,36
	PAS máx (Hgmm)	172±23	157±26	178±17	171±24	163±25	168±29	170±22	165±26	0,47
	PAD máx (Hgmm)	76±11	75±6	77±5	71±14	78±12	75±8	77±10	74±10	0,78
**Bioquímica sanguínea**										
	Hb (g/L)	123±11	125±4	133±12	125±18	136±21	135±19	131±16	129±16	0,69
	Sódio (mmol/L)*	139±3	138±4	141±3	141±2	141±2	142±2	140±3	140±3	0,86
	Potássio (mmol/L)	4,2±0,7	4,3±0,8	4,1±0,3	4,3±0,4	4,4±0,3	4,2±2,2	4,2±0,4	4,2±0,5	0,48
	Creatinina (µmol/L)	131±35	123±38	96±24	218±308	132±104	132±112	121±71	158±187	0,40
	Colesterol total (mmol/L)	4,6±1,6	4,6±1,2	4,0±1,0	4,0±1,0	4,5±0,8	4,3±0,7	4,3±0,5	4,2±0,9	0,95
	Triglicerídeos (mmol/L)	1,5±0,8	1,5±1,1	1,9±0,6	2,6±1,8	2,1±1,4	1,6±0,9	1,9±1,0	1,9±1,3	0,41
	Glicose (mmol/L)	7,5±4,0	6,2±1,3	6,4±1,0	7,7±3,3	6,1±1,2	5,3±1,4	6,6±2,2	6,4±2,4	0,27

Os valores são apresentados como média ± DP ou número de pacientes (porcentagens); GLM: modelo linear generalizado;

* diferença de grupo (HOSPITAL vs CASA): p=0,017. IMC: índice de massa corporal; FC: frequência cardíaca; PAS: pressão arterial sistólica; PAD: pressão arterial diastólica; Hb: hemoglobina; MET: equivalente metabólico.

Para os pacientes que decidiram treinar na academia do hospital, as sessões de exercícios foram realizadas sob a supervisão de um cinesiologista 3 dias/semana. Para os pacientes que treinaram em uma academia comunitária ou em casa, houve uma visita inicial no hospital durante a qual os pacientes receberam uma tabela de prescrição descrevendo o programa de treinamento e foram ensinados a realizar cada exercício, dependendo de quais dispositivos eles tinham acesso (ou seja, elásticos, pesos livres e/ou peso corporal), e de como controlar a intensidade durante as sessões de exercício (ou seja, familiarização com uma escala de esforço percebido). Se os pacientes estivessem se exercitando na academia comunitária, este documento era compartilhado com um treinador local. Se os pacientes estivessem treinando em casa, eles guardavam esse documento para si. Nos dias em que não havia treinamento, todos os pacientes foram solicitados a se manterem ativos caminhando pelo menos 30 minutos por dia em uma intensidade de 2-3/10 na escala de Borg.

As consultas de acompanhamento por telefone foram realizadas a cada quatro semanas para os pacientes que decidiram se exercitar fora do contexto hospitalar, a fim de manter a motivação e capturar a adesão ao programa. Para os pacientes que completaram o programa, uma segunda avaliação cardiovascular foi realizada aos seis meses.

### Dados extraídos de prontuários médicos

Os seguintes parâmetros foram extraídos dos prontuários dos pacientes que completaram o programa:

– Características clínicas: demografia, órgão transplantado, data do transplante e motivo do transplante;
– Avaliação cardiovascular na linha de base e seis meses depois: dados clínicos (peso (Health O Meter, modelo 500 KL) altura, circunferência da cintura, pressão arterial, frequência cardíaca (GE Caso T2100)); e dados biológicos (isto é, eletrólitos, Hb, perfil lipídico e glicemia);
– Avaliação da capacidade de exercício: os resultados foram extraídos dos laudos do teste de esforço realizado em esteira (esteira e ECG: GE Case T2100). O equivalente metabólico máximo (MET) foi determinado como o último estágio concluído no protocolo de Cornell. A FC máx foi determinada como a frequência cardíaca máxima atingida no pico do teste;
– Adesão ao programa de treinamento autorrelatada ou relatada pelo educador físico e responsável pelo programa.

### Análise de dados

Os resultados foram expressos em média e desvio padrão (DP), ou em número de casos e proporções (%), total e de acordo com os grupos (academia hospitalar, academia comunitária ou em casa). Todo o conjunto de dados foi selecionado para outliers para garantir a representatividade do grupo. O tamanho do efeito g de Hedge foi calculado para os principais desfechos:^27^ tamanhos de efeito entre 0,2 e 0,49 foram considerados efeito pequeno; entre 0,50 e 0,79 moderado; e superior a 0,8 efeito alto. A normalidade da distribuição foi analisada por meio de estatística descritiva (média, desvio padrão, mediana e amplitude), além de inspeções visuais. Modelos Lineares Generalizados (GLM) foram usados para comparar grupos e tempo (visita 1 vs. visita 2) devido ao pequeno tamanho da amostra. Um teste t pareado foi usado para comparar os valores pré e pós para todo o grupo (n=22). Os cálculos do tamanho da amostra não foram realizados, pois analisamos toda a coorte de pacientes e estamos apresentando os resultados de um estudo piloto. A significância estatística foi estabelecida em um nível alfa de 0,05 e todas as análises foram realizadas usando SPSS versão 24 (Chicago, IL, EUA).

## Resultados

**Figura 1:**
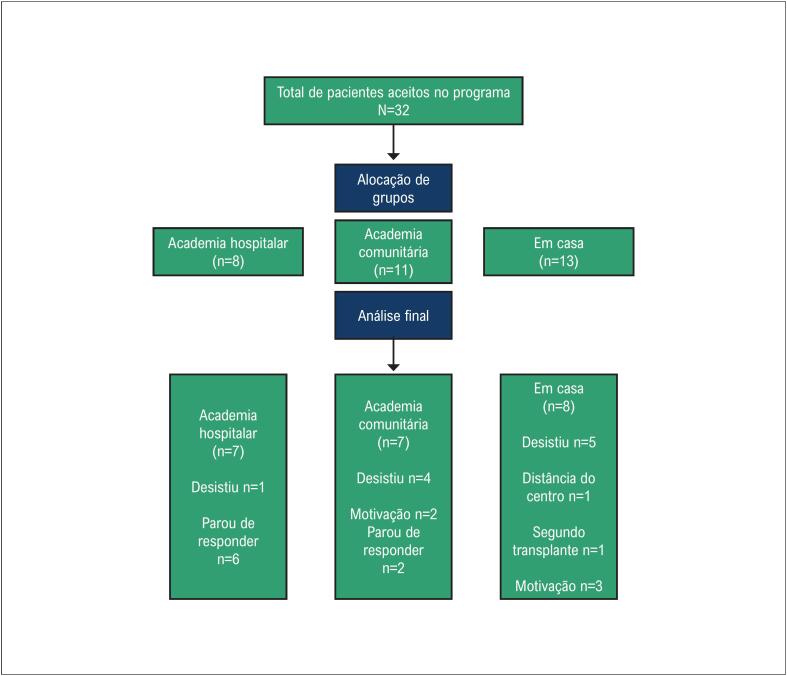
Fluxograma do estudo.

Dos primeiros 32 receptores de transplante que concordaram em participar deste programa de RBE, 10 (academia hospitalar n=1; academia comunitária n=4; e em casa n=5) não completaram seu programa (para detalhes, ver fluxograma – Figura 1): oito por desinteresse ou desmotivação, um por distância para ir ao centro para avaliação final e um por alteração do quadro clínico com necessidade de segundo transplante. A taxa de retenção foi de 69%. Entre os 22 pacientes que completaram o programa RBE, 7 treinaram na academia do hospital, 7 na academia comunitária e 8 em casa. A Tabela 1 descreve as características clínicas desses 22 pacientes. O GLM não mostrou diferenças para fator de grupo, visita (pré e pós) ou fatores de interação.

**Figura 2:**
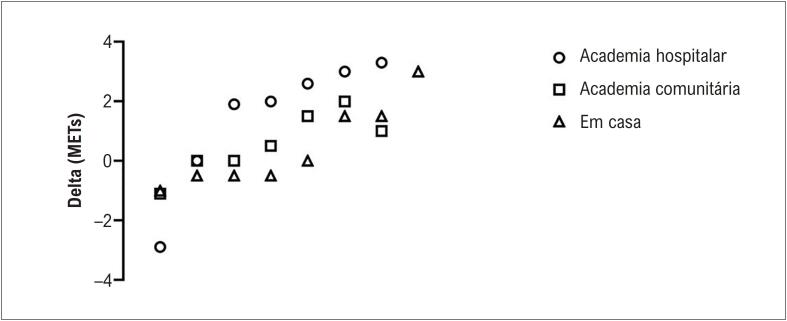
Mudanças individuais dos pacientes (deltas) em METs máximos de acordo com o grupo de treinamento físico. MET: equivalente metabólico.

**Figura 3:**
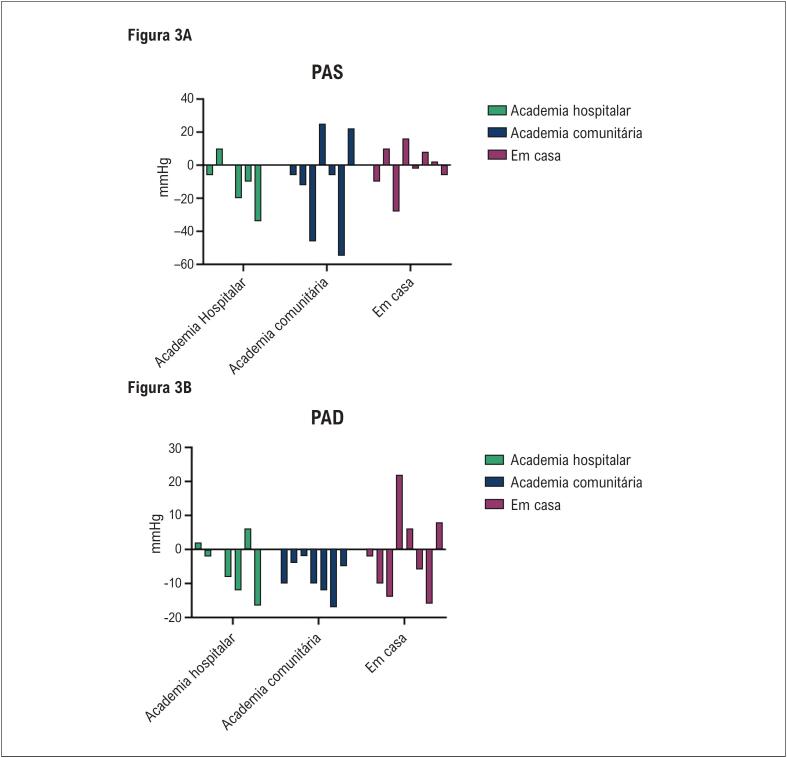
Mudanças (deltas) dos pacientes individuais na PAS (A) e PAD (B) de acordo com o grupo de treinamento físico. PAS: pressão arterial sistólica; PAD: pressão arterial diastólica.

Quando os resultados pré-pós foram analisados como um único grupo (n=22), encontramos significância para a pressão arterial diastólica (teste T - p= 0,037) e significância limítrofe para METs máx (teste T - p =0.072). As Figuras 2 e 3 descrevem os dados de pacientes individuais de valor delta para METs (Figura 2), pressão arterial sistólica (Figura 3A) e diastólica (Figura 3B).

Os parâmetros do teste de esforço são mostrados na Tabela 2. Os METs máx. gerais foram aumentados em 11,4% (g de Hedges = 0,39). Para aqueles que treinam na academia do hospital, os METs máx aumentaram 25,5% (Hedges’ g= 0,58), enquanto os METs máx aumentaram 10% (Hedges’ g= 0,25) para os pacientes treinando em uma academia comunitária, e 6,5% (Hedges’ g= 0,20) para aqueles em treinamento em casa. A Figura 1 apresenta análises delta individuais para METs.

Considerando todos os grupos juntos, a pressão arterial sistólica diminuiu 5,4% (g de Hedges = 0,49) e a pressão arterial diastólica diminuiu 5,2% (g de Hedges = 0,52). Os tamanhos de efeito de Hedges para as pressões arteriais sistólica e diastólica foram g= 0,51 e 0,40 para aqueles que treinavam na academia do nosso hospital; g= 0,60 e 1,15 para os que treinam em academia comunitária; eg= 0,07 e 0,10 para os que treinam em casa.

Nenhum evento adverso relacionado foi relatado durante o acompanhamento desses pacientes. Os educadores físicos responsáveis por esses pacientes não observaram diferenças entre os grupos quanto à conformidade e à adesão à prescrição de exercícios.

## Discussão

Um programa de RBE em receptores de transplante de rim e fígado parece ser seguro e tem benefícios na capacidade de exercício e fatores de risco cardiovascular, independentemente de como o programa é realizado. No entanto, a magnitude desses benefícios parece ser maior nos pacientes que treinam na academia do hospital em comparação com os demais (embora isso também possa refletir o viés de autosseleção do paciente).

A *Canadian Association for Cardiovascular Prevention and Rehabilitation* recomenda, como indicador de qualidade de programas de reabilitação, que a capacidade funcional deve aumentar por meio MET até o final da intervenção.^28,29^ Isso foi alcançado por 61% de nossos pacientes (n=6 em academia hospitalar, n=4 em academia comunitária e n=3 em casa). Além disso, 77% de nossos pacientes conseguiram manter sua capacidade de exercício ao longo dos 6 meses. Observamos benefícios semelhantes nas pressões arteriais sistólica e diastólica, embora nossos receptores de transplante renal estivessem teoricamente em maior risco de desenvolver hipertensão pós-transplante.^30^

A literatura sobre o treinamento físico em RTOS é escassa, revisões anteriores da literatura^31^ e uma meta-análise de ensaios clínicos randomizados^17^ não mostraram efeito sobre a capacidade de exercício para receptores de rim^32^ (apenas um estudo) ou fígado^33,34^ (apenas dois estudos). No entanto, estudos anteriores foram concebidos como programas totalmente supervisionados.^21^

A adesão a qualquer tipo de tratamento tem efeito direto na sua eficácia.^35–37^ Não haverá alta adesão a um programa de RBE se o paciente não expressar uma forte motivação para começar. No contexto específico dos RTOS, as preferências do paciente devem ser levadas em consideração, especialmente em relação à forma como o programa será entregue. Apesar disso, 31% dos nossos RTOS não concluíram o programa, especialmente aqueles que treinaram em uma academia comunitária ou em casa. Isso sugere que o acompanhamento por telefonemas regulares não é suficiente para manter nossos pacientes motivados e engajados. Considerando o desenvolvimento exponencial de plataformas e aplicativos web amigáveis para pacientes com RTOS,^38^ o próximo passo é construir recursos que ajudem a monitorar programas de exercícios - acreditamos que essas tecnologias podem ser a peça que faltava para programas realizados fora do contexto hospitalar.

### Limitações

Os resultados aqui apresentados são de uma análise retrospectiva da vida real, não de um estudo controlado randomizado, portanto, observa-se alguma flexibilização do rigor científico. Não avaliamos rigorosamente os fatores específicos que influenciam a escolha do paciente pelo tipo de RBE ou descontinuação do programa. O efeito da RBE na qualidade de vida desses pacientes não foi medido prospectivamente e nossa avaliação de adesão é limitada aos autorrelatos de pacientes e educadores físicos. O pequeno tamanho de nossa amostra prejudicou nossa análise e não nos permitiu provar que nossas descobertas, usando a analise de tamanho de efeito de Hedges, provavelmente não eram devidas ao acaso. Independentemente disso, a maioria de nossos pacientes conseguiu pelo menos manter a capacidade de exercício ao longo dos 6 meses. Além disso, este é o primeiro estudo que investigou o efeito de um programa de RBE focado na reabilitação de fase 3 (ou seja, não após a cirurgia), onde os pacientes já estão estáveis e é esperado algum declínio (não melhora) na função física. Ainda assim, o fato de sermos os primeiros a demonstrar os efeitos positivos do treinamento fora do hospital em RTOS também é encorajador.

## Conclusão

Programas de RBE em receptores de transplante de rim e fígado são viáveis e parecem fornecer resultados positivos na capacidade de exercício e nos fatores de risco cardiovasculares de risco clássicos. Devem ser incentivados, mesmo que sejam realizados fora do contexto hospitalar, pois a segurança parece assemelhar-se à do ambiente hospitalar. No entanto, os programas realizados em uma academia comunitária ou em casa devem ser associados a um telemonitoramento reforçado de cada paciente para garantir a adesão adequada e reduzir o risco de desmotivação e desengajamento.

## *Material suplementar

Para informação adicional, por favor, clique aqui


