# Supplementary material for: Implementação Clínica de Diferentes Estratégias para Reabilitação Baseada em Exercícios em Receptores de Transplante de Rim e Fígado: Um Estudo Piloto
Source: Arq Bras Cardiol. 2022 Jul 29;119(2):246–54. [Article in Portuguese] doi: 10.36660/abc.20210159 (PMC9363074; doi:10.36660/abc.20210159)
Supplement: Supplementary file 1 [file 2021-0159-supplemental.pdf]

Supplemental Table 1: General list of exercises used in resistance training

Exercise	Primary muscle(s)	Zone focus
Alternating Heel Touch	Abdominals - Obliques	Core
Crunch	Abdominals - Upper	Core
Crunch	Abdominals - Upper	Core
Sit-Up	Abdominals - Upper	Core
Lat Pulldown	Back - Latissimus Dorsi	Upper body
Pull-Up	Back - Latissimus Dorsi	Upper body
Bent-Over Dumbbell Row	Back - Lat.Dorsi/Rhomboids	Upper body
Bent-Over Hammer Dumbbell Row	Back - Lat.Dorsi/Rhomboids	Upper body
Machine Row	Back - Lat.Dorsi/Rhomboids	Upper body
One Arm Dumbbell Row	Back - Lat.Dorsi/Rhomboids	Upper body
Seated Cable Row	Back - Lat.Dorsi/Rhomboids	Upper body
Seated Row	Back - Lat.Dorsi/Rhomboids	Upper body
Bicep Curl	Biceps	Upper body
Dumbbell Curl	Biceps	Upper body
Hammer Curl	Biceps	Upper body
Seated Dumbbell Curl	Biceps	Upper body
Calf Press	Calves - Gastrocnemius	Lower body
Dumbbell Calf Raise	Calves - Gastrocnemius	Lower body
Chest Press	Chest – Pectoralis	Upper body
Dumbbell Bench Press	Chest – Pectoralis	Upper body
Push-Up	Chest – Pectoralis	Upper body
Dumbbell Fly	Chest – Pectoralis	Upper body
Pec Deck Fly	Chest – Pectoralis	Upper body

Hip Lift	Legs – Hamstrings	Lower body
Leg Curl	Legs – Hamstrings	Lower body
Dumbbell Lunge	Legs – Quadriceps	Lower body
Dumbbell Squat	Legs – Quadriceps	Lower body
Leg Press	Legs – Quadriceps	Lower body
Lunge	Legs – Quadriceps	Lower body
Step-Up	Legs – Quadriceps	Lower body
Leg Extension	Legs – Quadriceps	Lower body
Superman	Lower Back - Erector Spinae	Core
Dumbbell Front Raise	Shoulders - Delts/Traps	Upper body
Dumbbell Lateral Raise	Shoulders - Delts/Traps	Upper body
Seated Dumbbell Rear Delt Raise	Shoulders - Delts/Traps	Upper body
Dumbbell Kickback	Triceps	Upper body
Lying Tricep Extensions	Triceps	Upper body
Tricep Extensions	Triceps	Upper body

Supplemental Table 2: Prescription table of our EBR program for SOTR

Patients' profile	Stage	Aims/Intensity	Session duration	Frequency
Very low physical function	Initiation Week 0-4	AT: 50 to 80%<br>RT: 2x 12-15 reps<br>RPE 4-5/10	60 min/day	2-3 sessions/week
	Maintenance Week >4	AT: 50 to 80/85%<br>RT: 2x12-15 reps<br>RPE 5-7/10	60 min/day	3 sessions/week
Appropriate physical function	Initiation Week 0-4	AT: 50 to 80%<br>RT: 2x12-15 reps<br>RPE 5-7/10	60 min/day	3 sessions/week
	Maintenance Week >4	AT: 50 to 85%<br>RT: 2-3x 12-15 reps<br>RPE 5-7/10	60 min/day	3 sessions/week

EBR: exercise-based rehabilitation; RPE: rate of perceived exertion; AE: aerobic training; RT: resistance training.
