# Supplementary material for: Implementação Clínica de Diferentes Estratégias para Reabilitação Baseada em Exercícios em Receptores de Transplante de Rim e Fígado: Um Estudo Piloto
Source: Arq Bras Cardiol. 2022 Jul 29;119(2):246–54. [Article in Portuguese] doi: 10.36660/abc.20210159 (PMC9363074; doi:10.36660/abc.20210159)
Supplement: Translation

# Translation: Clinical Implementation of Different Strategies for Exercise-Based Rehabilitation in Kidney and Liver Transplant Recipients: A Pilot Study

**Authors:** Paula A. B. Ribeiro, Mathieu Gradassi, Sarah-Maude Martin, Jonathan Leenknegt, Mathilde Baudet, VyVan Le, Marie-Pascale Pomey, Agnes Räkel, François Tournoux

**Affiliations:** 1 Quebec Canada Unité de recherche @coeurlab – Centre de Recherche du Centre Hospitalier de l’Université de Montréal (CRCHUM), Quebec – Canada; 2 Quebec Canada Centre de Cardiologie Preventive du Centre Hospitalier de l’Université de Montréal, Quebec – Canada; 3 Québec Canada Département des sciences de l’activité physique, Université du Québec à Montréal, Québec – Canada; 4 Québec Canada Département de Cardiologie du Centre Hospitalier de l’Université de Montréal, Québec – Canada; 5 Québec Canada École de santé publique, Université de Montréal, Québec – Canada; 6 Québec Canada Département d’Encrinologie du Centre Hospitalier de l’Université de Montréal, Québec – Canada

**Keywords:** Exercise, Exercise Movement Techniques, Physical Conditioning Human, Kidney/transplantation, Liver/transplantation, Exercise Therapy

## Abstract

**Background:**

Cardiovascular disease is among the leading causes of death in solid organ transplant recipients with a functional graft. Although these patients could theoretically benefit from exercise-based rehabilitation (EBR) programs, their implementation is a challenge.

**Objective:**

We present our initial experience on different delivery modes of a pilot EBR program in kidney and liver transplant recipients.

**Methods:**

Thirty-two kidney or liver transplant recipients were invited for a 6-month EBR program delivered at the hospital gym, community gym or at home, according to the patient’s preference. The significance level adopted was 5%.

**Results:**

Ten patients (31%) did not complete their program. Among the 22 who did, 7 trained at the hospital gym, 7 at the community gym, and 8 at home. The overall effect was an 11.4% increase in maximum METs (Hedges’ effect size g = 0.39). The hospital gym group had an increase in METs of 25.5% (g= 0.58, medium effect size) versus 10% (g= 0.25), and 6.5% (g= 0.20) for the community gym and home groups, respectively. There was a beneficial effect on systolic and diastolic blood pressures, greater for the hospital gym (g= 0.51 and 0.40) and community gym (g= 0.60 and 1.15) groups than for the patients training at home (g= 0.07 and 0.10). No significant adverse event was reported during the follow-up.

**Conclusion:**

EBR programs in kidney and liver transplant recipients should be encouraged, even if they are delivered outside a hospital gym, since they are safe with positive effects on exercise capacity and cardiovascular risk factors.

## Introduction

Short-term survival among solid-organ transplant recipients (SOTRs) has significantly improved due to decreased mortality from infections and acute graft rejections.^1^ Although liver and kidney transplant recipients have a lower cardiovascular (CV) risk than their counterparts on transplant waiting lists,^2,3^ their mortality risk is still higher than the general population.^4,5^ In fact, cardiovascular diseases are the most common causes of death in patients with a functional graft and are responsible for 30% of early graft loss after kidney transplantation.^4,6^ Certain pre-transplantation risk factors, including diabetes, hypertension, dyslipidemia, and obesity, contribute to this high CV risk.^6,7^ There are also post-transplantation factors which contribute to this CV risk, such as a new onset of diabetes,^8^ development of a metabolic syndrome^9^ and sedentary lifestyle.^10^ Most of the SOTRs do not achieve the guideline-recommended levels of physical activity in their daily routine^11,12^ suggesting that patients could benefit from more guidance and tailored social and professional support^13,14^ to improve their daily physical activity.

Since exercise-based rehabilitation programs (EBR) improve cardiovascular risk factors in the general population,^6,15^ they are expected to have a beneficial impact on solid organ transplant recipients. While the effects of these programs are well known in cardiac and lung transplant recipients (due to direct effects of exercise on cardiac and pulmonary function),^16–19^ their benefits and safety are more uncertain for other SOTRs patients.^17,18,20,21^ Costs, logistics, and insurance coverage are also significant barriers^22^ which limit the rapid and widespread implementation of these EBR programs for this specific population. We believe that tailored delivery strategies and out-center programs could be helpful to overcome these challenges and enroll patients that would not participate in centre-based programs, especially in unexpected situations such as the COVID-19 pandemic.^23,24^

Therefore, we present our initial experience on the cardiovascular effects of different delivery modes of a pilot EBR program in kidney and liver transplant recipients.

## Methods

In 2016, we conducted at our institution a randomized pilot study on the impact of resistance training on factors involved in the development of new-onset diabetes after renal transplantation.^20^ We learned from this study that almost 55% of our patients declined the invitation to join because they were unable to come to our center as often as required by this program (3 times a week). Our team decided to design a new EBR program for kidney and liver transplant patients which can be delivered at the hospital gym as well as in a community gym or at home, depending on the patient’s preference. We present here our initial experience with the first 32 patients engaged within this new Combined EBR program. This retrospective analysis was approved by the CRCHUM Ethical Committee on Human Research, which complies with the Declaration of Helsinki (REC 2017-6733).

### The EBR Program

In our institution, SOTRs (18 years and older) are invited to join the EBR program after transplantation as part of their care trajectory, usually 6 months after renal transplantation and 9 months after liver transplantation. All kidney and liver-recipient patients that participated in our program between 2016 to 2018 were included in our analysis. Pre-participation assessment (physical exam and stress test) was performed at the hospital by a cardiologist and a kinesiologist. In the absence of cardiovascular contraindication, each patient participated in a 6-month EBR, tailored according to its current condition and whether or not they preferred to train outside the hospital context. A discussion between the patient and the kinesiologist on the pros and cons was made at that time. The exercise prescription followed the ACSM and CAN-Restore recommendations,^25,26^ combining aerobic, resistance and flexibility exercises: 1) aerobic training: 3-5 times a week, targeting 50-80% VO_2_max (5-6 Borg), starting with 20 min/section and increasing progressively up to 60 min; 2) resistance training: 2-3 times a week, 1-3 series of 10 to 15 repetitions of 5-6 exercises (total of 20 to 30 min), using multi-joints exercises including the major muscle groups according to patient’s abilities (the full list of the prescribed exercises is available in supplemental material – Table 1); and 3) flexibility exercises 2-3 times a week, 2-3 exercises/positions according to patient symptom limitation (i.e., pain). The prescription table is available in the supplemental material – Table 2.

**Table 1:** Clinical characteristics according to the intervention group

		Hospital gym (n=7)	Community gym (n=7)	Home-based (n=8)	Total (n=22)
Age		58.0±6.9	53.7±12	60.4±8.0	57.5±9.2
Gender (M/F)		5/2	3/4	6/2	14/8
Transplant time (months)		126±97	103±71	113±93	114±84
Range transplant time (min-max)		253 (5 - 258)	198 (8 - 206)	242 (12 – 254)	253 (5 - 258)
**Transplant (n)**					
	Kidney	2	6	7	15
	Liver	3	1	0	4
	Kidney+Pancreas	2	0	1	3
Diabetes		2	5	3	10
Hypertension		4	5	6	15
**Medication use**					
	Beta-blocker	2	6	3	11
	Immunossupressors	4	4	7	15

Values are presented as mean ± SD or numbers of patients (percentages); GLM: generalized linear model;

* group difference (CHUM vs HOME): p=0.017.

**Table 2:** Clinical characteristics according to the intervention group

		Hospital gym (n=7)		Community gym (n=7)		Home-based (n=8)		TOTAL (n=22)		Interaction GLM
		Pre	Post	Pre	Post	Pre	Post	Pre	Post	
Weight (Kg)		81.3±18.9	81.3±20.4	91.4±14.7	85.3±15.1	82.9±12.0	80.3±13.9	85.1±15.2	82.1±16.2	0.87
BMI (m/kg^2^)		28.6±5.8	28.6±6.4	32.1±4.8	30.1±2.5	30.1±4.7	29.1±4.6	30.2±5.1	29.2±4.7	0.86
Waist circumference (cm)		100.4±15.5	98.9±16.1	111.8±11.7	105.0±8.6	105.7±8.4	105.0±9.2	105.7±12.7	102.6±12.2	0.78
**Exercise test**										
	METs max	5.5±2.3	6.9±2.2	6.0±2.0	6.6±2.4	6.1±1.7	6.5±2.0	5.8±1.9	6.6±2.1	0.76
	METs predicted (%)	75±28	96±31	81±34	87±37	91±35	96±40	82±32	93±35	0.76
	VO_2_max calculated (ml.kg.min^−1^)	19.2±7.9	24.1±7.8	21.1±7.0	23±8.3	21.2±6.0	22.8±7.1	20.5±6.7	23.3±7.4	0.76
	Exercise time (min)	7:47±3:51	8:11±3:21	6:00±1:31	7:00±1:37	7:37±2:36	7:30±2:55	7:09±2:47	7:33±2:39	0.86
	HR max (bpm)	133±18	131±35	131±33	130±35	131±26	130±25	132±25	130±30	0.99
	HR predicted (%)	82±12	80±23	78±20	77±19	80±14	81±17	80±15	79±18	0.98
	SBP pre-test	131±15	122±18	138±20	127±14	125±16	124±9	131±17	124±10	0.55
	DBP pre-test*	74±8	71±6	81±6	73±7	76±8	75±10	77±8	73±7	0.36
	SBP max (Hgmm)	172±23	157±26	178±17	171±24	163±25	168±29	170±22	165±26	0.47
	DBP max (Hgmm)	76±11	75±6	77±5	71±14	78±12	75±8	77±10	74±10	0.78
**Blood analysis**										
	Hb (g/L)	123±11	125±4	133±12	125±18	136±21	135±19	131±16	129±16	0.69
	Sodium (mmol/L)*	139±3	138±4	141±3	141±2	141±2	142±2	140±3	140±3	0.86
	Potassium (mmol/L)	4.2±0.7	4.3±0.8	4.1±0.3	4.3±0.4	4.4±0.3	4.2±2.2	4.2±0.4	4.2±0.5	0.48
	Creatinine (µmol/L)	131±35	123±38	96±24	218±308	132±104	132±112	121±71	158±187	0.40
	Total cholesterol (mmol/L)	4.6±1.6	4.6±1.2	4.0±1.0	4.0±1.0	4.5±0.8	4.3±0.7	4.3±0.5	4.2±0.9	0.95
	Triglycerides (mmol/L)	1.5±0.8	1.5±1.1	1.9±0.6	2.6±1.8	2.1±1.4	1.6±0.9	1.9±1.0	1.9±1.3	0.41
	Glucose (mmol/L)	7.5±4.0	6.2±1.3	6.4±1.0	7.7±3.3	6.1±1.2	5.3±1.4	6.6±2.2	6.4±2.4	0.27

Values are presented as mean ± SD or numbers of patients (percentages); GLM: generalized linear model;

* group difference (CHUM vs HOME): p=0.017 BMI: body mass index; HR: heart rate; SBP: systolic blood pressure; DBP: diastolic blood pressure; Hb: hemoglobin; MET: metabolic equivalent.

For patients who decided to train at our hospital gym, exercise sessions were performed under the supervision of a kinesiologist 3 days/week. For patients training at a community gym or at home, there was an initial visit at the hospital during which the patients received a table of prescription describing the training program and is taught how to perform each exercise, depending on which devices they have access to (i.e. elastics, free weights and/or bodyweight), and how to control the intensity during exercise sessions (i.e. familiarization with a perceived exertion scale). If patients were exercising at the community gym this document was shared with a local trainer. If the patients were training at home, they kept this document for themselves. During the days when no training was scheduled, all the patients were asked to keep themselves active by walking at least 30 minutes per day at an intensity of 2-3/10 on the Borg scale.

Follow-up consultations by phone were performed every four weeks for patients who decided to exercise outside the hospital context in order to maintain motivation and to capture program compliance. For patients who completed the program, a second cardiovascular assessment was performed at six months.

### Abstracted data from medical records

The following parameters were extracted from the medical records of the patients who completed the program:

– Clinical characteristics: demographics, transplanted organ, date of transplantation and reason for transplantation;
– Cardiovascular assessment at baseline and six months: clinical data (weight (Health O Meter, model 500 KL) height, waist circumference, blood pressure, heart rate (GE Case T2100)); and biological data (i.e. electrolytes, Hb, lipid profile and glycemia);
– Exercise capacity assessment: results were extracted from the reports of the stress test performed on a treadmill (treadmill and ECG: GE Case T2100). The maximum metabolic equivalent (MET) was determined as the last completed stage on the Cornell protocol. HR max was determined as the maximum heart rate achieved at the peak of the test;
– Patient self-reported or kinesiologist-reported adherence to the training program.

### Data analysis

Results were expressed as means and standard deviation (SD), or as the number of cases and proportions (%), total and according to groups (hospital gym, community gym or home-based). The entire dataset was screened for outliers to ensure group representativeness. Hedges’ g effect size was calculated for main outcomes:^27^ effect sizes between 0.2 and 0.49 are considered small effect; between 0.50 and 0.79 moderate; and higher than 0.8 high effect. Distribution normality was analyzed using descriptive statistics (mean, standard deviation, median and range), as well as visual inspections. Generalized Linear Models (GLM) were used to compare groups and time (visit 1 vs. visit 2) due to small sample sizes. A paired t-test was used to compare pre- and post-values for the whole group (n=22). Sample size calculations were not performed since we analyzed the entire cohort of patients, and we are presenting the results in a pilot analysis fashion. Statistical significance was set at an alpha level of .05, and all analyses were performed using SPSS version 24 (Chicago, IL, USA).

## Results

From the first 32 transplant recipients who agreed to participate in this EBR program, 10 (hospital gym n=1; community gym n= 4; and home-based n=5) did not complete their program (for details, see flowchart – Figure 1): eight due to lack of interest or motivation, one due to distance to go to the centre for final assessment, and one due to a change in his medical condition with the need of a second transplantation. The retention rate was 69%. Among the 22 patients who completed the EBR program, 7 trained at the hospital gym, 7 at a community gym and 8 at home. Table 1 describes the clinical characteristics of those 22 patients. GLM did not show any differences for group factor, visit (pre and post) or interaction factors.

**Figure 1:** Flowchart of the study.

When the pre-post results were analyzed as one single group (n=22), we found significance for the diastolic blood pressure (T-test - p= 0.037) and borderline significance for METs max (T-test - p = 0.072). Figures 2 and 3 describe delta-value individual patient data for METs (Figure 2), systolic (Figure 3A) and diastolic blood pressure (Figure 3B).

**Figure 2:** Individual patients’ changes (deltas) in maximal METs according to exercise training group. MET: metabolic equivalent.

**Figure 3:** Individual patients’ changes (deltas) in SBP (A) and DBP (B) according to exercise training group. SBP: systolic blood pressure; DBP: diastolic blood pressure.

Exercise test parameters are shown in Table 2. Overall METs max was increased by 11.4% (Hedges’ g= 0.39). For those training at the hospital gym, METs max increased by 25.5% (Hedges’ g= 0.58), whereas METs max increased by 10% (Hedges’ g= 0.25) for patients training at a community gym, and 6.5% (Hedges’ g= 0.20) for those in home-based training. Figure 1 presents individual delta analyses for METs.

Considering all groups together, systolic blood pressure decreased by 5.4% (Hedges’ g= 0.49), and diastolic blood pressure decreased by 5.2% (Hedges’ g= 0.52). Hedges’ effect-sizes for systolic and diastolic blood pressures were g= 0.51 and 0.40 for those training at our hospital gym; g= 0.60 and 1.15 for those training at a community gym; and g= 0.07 and 0.10 for those training at home.

No related adverse event was reported during the follow-up of these patients. The kinesiologists in charge of these patients did not observe any differences between groups in terms of compliance and adherence to exercise prescription.

## Discussion

An EBR program in kidney and liver transplant recipients appears to be safe and has benefits on exercise capacity and cardiovascular risk factors, regardless of how the program is delivered. However, the magnitude of these benefits seems to be greater in patients training at the hospital gym compared to the other ones (though this may reflect patient self-selection bias as well).

The Canadian Association for Cardiovascular Prevention and Rehabilitation recommends, as a Quality Indicator of rehabilitation programs, that functional capacity should increase by a half MET through the end of intervention.^28,29^ This was attained by 61% of our patients (hospital-based n=6, community-based n=4, and home-based n=3). Moreover, 77% of our patients were able to maintain their exercise capacity over the course of the 6-months. We observed similar benefits on systolic and diastolic blood pressures, although our kidney transplant recipients were theoretically at a higher risk of developing post-transplant hypertension.^30^

The literature about exercise training in SOTRs is scarce, and previous reviews of the literature^31^ and a meta-analysis of randomized controlled trials^17^ showed no effect on exercise capacity for kidney^32^ (only one study) or liver recipients^33,34^ (only two studies). However, previous trials have been designed as fully supervised programs.^21^

Compliance with any kind of treatment has a direct effect on its efficacy.^35–37^ There will not be high compliance to an EBR program if the patient does not express a strong motivation to begin with. In the specific context of SOTRs, patient preferences have to be taken into consideration, especially regarding how the program is going to be delivered. Despite that, 31% of our SOTRs did not complete their program, especially among those training in a community gym or at home. This suggests that follow-up by regular phone calls is not sufficient to keep our patients motivated and engaged. Considering the exponential development of user-friendly web platforms and apps for SOTR patients,^38^ the next step is to build up features that help monitor exercise programs - we believe these technologies could be the missing piece for these programs delivered outside the hospital context.

### Limitations

The results presented here are from a real-life setting retrospective analysis, not a randomized controlled trial, therefore some flexibilization of the scientific rigor is observed. We did not rigorously assess the specific factors influencing the patient’s choice of the type of EBR or discontinuation of the program. The effect of EBR on quality of life of these patients was not prospectively measured, and our compliance assessment is limited to patient and kinesiologist self-reports. Our small sample size underpowered our analysis and did not allow us to prove that our findings using Hedges’ effect size method were not likely to be due to chance. Regardless, most of our patients were able to at least maintain exercise capacity over the course of the 6-months. Moreover, this is the first study that investigated the effect of an EBR program that is focused on phase 3 rehabilitation (i.e. not after surgery), where patients are already stable and some decline (not improvement) in physical function is expected. Still, the fact that we are the first to demonstrate the positive effects of out-of-center training in SOTR is also encouraging.

## Conclusion

EBR programs in kidney and liver transplant recipients are feasible and seem to provide positive results on exercise capacity and classic cardiovascular risk factors. They should be encouraged, even if they are delivered outside a hospital context, as safety seems to be similar to a hospital setting. However, programs delivered in a community gym or at home should be associated with a reinforced telemonitoring of each patient to ensure proper compliance and reduce the risk of demotivation and disengagement.

## *Supplemental Materials

For additional information, please, click here
